# A meta-analytic review of the associations between dimensions of religious coping and psychological symptoms during the first wave of the COVID-19 pandemic

**DOI:** 10.3389/fpsyt.2023.1097598

**Published:** 2023-01-19

**Authors:** Cecilia Cheng, Weijun Ying

**Affiliations:** ^1^Social and Health Psychology Laboratory, Department of Psychology, The University of Hong Kong, Hong Kong, Hong Kong SAR, China; ^2^Department of Education, Johns Hopkins University, Baltimore, MD, United States

**Keywords:** coping, stress, anxiety, mental health, public health, epidemic, depression, coronavirus (COVID-19)

## Abstract

**Introduction:**

In the first wave of the COVID-19 pandemic, the unknown etiology and treatment of the highly transmissible coronavirus posed considerable threats to public mental health. Many people around the globe turned to religion as an attempt to mitigate their heightened psychological distress, but mixed findings have been obtained regarding the association between the use of religious coping and two psychological symptoms—anxiety and depressive symptoms—widely reported in the initial wave.

**Objective:**

The present meta-analysis was conducted to resolve the empirical inconsistency by synthesizing this body of studies and identifying both individual and national-level factors that accounted for the inconsistent findings.

**Methods:**

Following PRISMA guidelines, the literature search and data screening procedures yielded 42 eligible studies, with 25,438 participants (58% females, average age = 36.50 years) from 24 countries spanning seven world regions.

**Results:**

Overall, the results showed that only negative religious coping was positively associated with psychological symptoms (*r* = 0.2886, *p* < 0.0001). Although the associations of both general and positive religious coping with psychological symptoms were non-significant (*r*s = 0.0425 and −0.0240, *p*s > 0.39), the moderation analysis revealed significant positive associations between positive religious coping and psychological symptoms in two demographic groups who experienced greater pandemic distress than their counterparts: younger participants and female participants.

**Discussion:**

This meta-analysis provides a nuanced understanding of the complex nature of religious coping in the initial wave of the COVID-19 pandemic when the levels of public anxiety and stress were heightened. The exclusive use of religious coping may not be associated with low levels of psychological symptoms, implying the importance of supplementing the deployment of this strategy with an array of other strategies. Therapists of mental health interventions should show their clients how to make good use of positive religious coping together with other strategies, and how to avoid the use of negative religious coping, to handle their psychological problems.

**Systematic review registration:**

https://osf.io/shb32/

## 1. Introduction

Since late 2019, the COVID-19 outbreak has swept swiftly across the globe, affecting more than 650 million people in 228 countries and territories worldwide as of December 2022 (1). As COVID-19 was caused by an atypical, novel type of coronavirus when it first emerged (2, 3), levels of public anxiety and stress were heightened in the initial wave of the pandemic from December 2019 to July 2020 (4–9). Many individuals from affected regions perceived a low sense of control or hope (10, 11) when unprecedented mandatory disease mitigation measures were implemented during this period (12–15).

During the outbreak of COVID-19, many individuals have resorted to religion for stress relief. Religion constitutes an important part of human civilization and exerts a huge impact on people’s behavior (16), and religious coping has been commonly adopted for handling stressful life circumstances, especially those whose outcomes are perceived to be largely beyond one’s control (17–20). Religious coping is defined as to the use of religious beliefs or behavior to prevent or alleviate the unpleasant emotions elicited by stressful life circumstances (21). Religious coping is proposed to serve five major functions, namely searching for meaning, achieving a sense of mastery and control, seeking comfort or spirituality, increasing intimacy with others, and transforming life (22).

When the World Health Organization declared COVID-19 a pandemic in March 2020, the search volume of the keyword “prayer” on Google search engine increased by 30% relative to all other Google searches, soaring to a record-high level (23). Some scholars have maintained that the use of religious coping to deal with pandemic stress should have mental health benefits, primarily because religion could serve as an alternative source of emotional support during these difficult times, when support from social network members was largely inaccessible because of physical distancing measures (24, 25). In line with these notions, some studies conducted during the initial wave of COVID-19 have shown that more frequent use of religious coping was associated with lower levels of perceived stress, better mental health, and lower sense of helplessness, and that less frequent use of religious coping was associated with higher levels of psychological symptoms such as anxiety and depression (26–29).

Despite such psychological benefits, there were also studies that yielded null or even contrary findings regarding the association between the use of religious coping and certain mental health criteria. Specifically, some studies have revealed that the frequency of using religious coping was unrelated to stress, anxiety, and depressive symptoms (30–32), whereas others have revealed that these coping–symptom associations were positive (33–35). For example, in a study conducted among individuals diagnosed with COVID-19, the use of religious coping was positively associated with death anxiety (36). Furthermore, Cowden et al. (37) found that greater use of religious coping was positively associated with perceived suffering and resource losses across physical, psychological, and interpersonal domains.

To resolve this body of inconsistent empirical evidence regarding the association between the use of religious coping and psychological symptoms during the first wave of the COVID-19 pandemic, a meta-analysis was conducted to systematically evaluate the findings derived from prior studies and to provide a quantitative estimate of the overall effect size of the religious coping–symptom association (38, 39). In addition, a moderator analysis was performed to explain possible between-study variations in the effect size estimates.

A review of the literature revealed that three clusters of moderators could account for the between-study differences. First, the dimension of religious coping has been proposed to moderate the magnitude of the coping–symptom association. Earlier research (40, 41) conceptualized religious coping as a non-specific unidimensional construct labeled “general religious coping” (GRC) (40, 42). Subsequent works have found that GRC could have both desirable and undesirable effects on mental health and thus have argued for the need to distinguish positive religious coping (PRC) from negative religious coping (NRC) (16, 43).

Typical examples of PRC are seeking psychological comfort from God or a transcendent force and seeing God as a partner who guides one along the journey of life (16, 43). From psychodynamic and developmental perspectives (44), religion has beneficial effects on mental health through emphasizing the inner and long-lasting meaning of life, cultivating a sense of God’s blessings, and providing personal and community resources to cope with stressors that are largely beyond one’s control. Studies have supported this notion by revealing the mental health benefits of PRC, such as the amelioration of depressive symptoms and the improvement of health-related quality of life (19, 20).

Typical examples of NRC are perceived conflicts and struggles with God or spirituality, difficulty seeing the meaning of life, doubts about support from God, and anger toward God (16, 43, 45). In this perspective, NRC does not only fail to relieve stress but can even elicit greater unpleasant emotions such as agitation and fury. This dimension of religious coping has been found to be positively associated with anxiety and depressive symptoms, as well as with poor health-related quality of life across various life domains (20, 46).

Second, the magnitude of the religious coping–symptom association may vary according to age and sex. Our notion stems from studies that have revealed that individuals of different sexes and ages tended to react to stress and to use religious coping in diverse ways. With regard to age differences, older people were found to be more religious (47, 48). Moreover, older patients with chronic hepatitis C tended to rely more on religion and to search for meaning as coping mechanisms to deal with stress more than their younger counterparts (49).

With regard to sex differences, studies have shown that the spouses of male patients with lung cancer tended to use religious coping to handle stress more than those of female patients (50). Similar sex differences in the use of religious coping were also obtained in research conducted during the COVID-19 pandemic (51). These findings may be explained by research showing that women are characterized by a higher level of religiosity than men (48, 52). In addition, female adolescents tended to use PRC more to cope with pandemic stress, whereas their male counterparts tended to use NRC more. Such sex differences may be explained by men’s greater tendency to see God as controlling (53, 54).

Third, the strength of the religious coping–symptom association was proposed to vary by two religion-related characteristics at the national level: dominant religion and religiosity. COVID-19 has affected many countries with various dominant religions, and thus, the religious beliefs and practices of residents of these countries should differ considerably. According to an extensive global analysis of religion (55), Christianity is the most widely practiced religion worldwide, and it is the dominant religion in many Western countries in North and South America, Western Europe, and Oceania (56). Islam and Hinduism are the second and third most widely practiced religions, respectively, and are the dominant religions in many non-Western countries in the Middle East and Asia (57, 58). In addition to the dominant religion, there are also vast between-country variations in the extent of national religiosity, which reflects the overall level of religiosity of the residents of a country (59). National religiosity comprises the key components of religious affiliation, attendance of religious activities, and religious beliefs (60, 61).

In multinational comparisons, the potential confounding effects of certain national characteristics should be controlled to ensure robust hypothesis testing. Socioeconomic development is a crucial confounding factor. This notion stems from a previous multinational comparison that identified differences in national subjective well-being among countries with distinct levels of socioeconomic development (62). Residents of developed (vs. developing) countries are generally less susceptible to psychological problems because of the availability of better social welfare and healthcare services.

A multinational comparison may also be confounded by the between-country differences in the number of confirmed COVID-19 infections and mortality cases (1) as well as the strictness of the COVID-19 containment measures imposed (63). During the first wave of the pandemic, some governments (e.g., Italy) issued stay-at-home orders with exceptions for “essential” trips such as clinic visits, while others (e.g., France) required their residents not to leave home with minimal exceptions (e.g., once per week or one household member at a time). However, some governments (e.g., Japan) only advised their residents not to leave home and did not issue any mandates.

In summary, the present meta-analysis aimed to quantitatively synthesize the literature investigating religious coping and mental health issues amid the first wave of the COVID-19 pandemic, and to account for potential between-study variations in the associations between religious coping and psychological symptoms by three clusters of moderators (i.e., dimension of religious coping, demographics, and religion-related characteristics).

## 2. Methods

The present meta-analysis was conducted to address the research questions regarding the hypothesized variations in the magnitude of the three dimensions of religious coping (i.e., GRC, PRC, and NRC) and two major indicators of psychological symptoms (i.e., depressive and anxiety symptoms) experienced during the initial wave of the COVID-19 pandemic. The meta-analysis was performed and reported in compliance with the Preferred Reporting Items for Systematic Reviews and Meta-Analysis (PRISMA) 2020 guidelines (64). As all the data were extracted from previous primary studies, no institutional ethical approval was required. The study design, eligibility criteria, coding process, and data-analytic procedures of the meta-analysis were preregistered *via* the Open Science Framework^1^.

### 2.1. Eligibility criteria

To screen potentially relevant eligible articles, eligibility criteria were developed *a priori*. Specifically, inclusion criteria were primary studies that included measures of both religious coping and any of the aforementioned target symptoms. Studies were excluded if at least one of the following criteria were met: (a) no empirical data (e.g., reviews and qualitative studies); (b) no relevant measures of religious coping or target symptoms; (c) data were collected before the COVID-19 pandemic; (d) duplicate samples; or (e) inadequate information for effect size estimation.

### 2.2. Information sources

Computerized literature searches were first conducted to systematically locate primary studies. These general and discipline-specific databases spanned the social science (e.g., Applied Social Sciences Index and Abstracts and PsycInfo^®^), health and medical science (e.g., Health and Safety Science Abstracts and MEDLINE^®^), and multi-disciplinary (e.g., Scopus and Web of Science) research fields. Moreover, gray literature (e.g., Google Scholar and OpenGrey) and dissertation databases (e.g., Open Access Theses and Dissertations and Networked Digital Library of Theses and Dissertations) were searched for additional primary studies, especially unpublished ones. Finally, scholars were contacted to request unpublished or unavailable works. Requests were made to 24 investigators, of whom 13 provided articles, data, or statistics.

### 2.3. Search strategy

When conducting the various literature searches, the following thesaurus text words and MeSH (Medical Subject Headings) were combined using the Boolean operators “AND” and “OR”: (“religious coping” OR “religion coping”) AND (“COVID” OR “coronavirus”) AND (“anxiety*” OR “depressi*” OR “mental health” OR “distress” OR “disorder”). These combined terms were searched in the title, abstract, and subject of potentially relevant works.

To obtain as many potentially relevant articles as possible, we did not impose any restrictions regarding the articles’ publication status, publication date, language, study design, or sample characteristics (i.e., country of origin, age group, race, and ethnicity).

### 2.4. Selection and data collection processes

As the search results were derived from multiple sources, duplicate records were identified and removed using the EndNote software (version 20.4.1). Then, two independent reviewers conducted preliminary relevance screening and perused the full-text articles for further screening and coding.

After the pool of eligible studies was finalized, the data extraction task was carried out by the same two reviewers according to a coding manual with full descriptions of the pre-established inclusion and exclusion criteria. The triangulation method was used to enhance inter-reviewer reliability (65). Specifically, the two reviewers were calibrated using a random sample of 10% of the eligible studies. Upon completion of the calibration process, each reviewer coded the remaining studies on their own. Any discrepancies between the reviewers were resolved with the assistance of the first author in *post-hoc* meetings.

Inter-reviewer reliability was evaluated using the concordance statistic of Krippendorff alpha (66). The results showed adequate inter-reviewer reliability for the final coding (Krippendorff alphas ≥ 0.69 for all the coded items).

### 2.5. Data items

Pearson *r* was chosen as the target effect size for the present meta-analysis because of its high interpretability (67), and the effect size indicated the magnitude of the association between a dimension of religious coping and psychological symptoms. If Pearson *r* was not reported in any eligible studies, relevant statistics (e.g., *t*-values and odds ratios) were transformed into Pearson *r* using the esc package written in the R programing language (68).

The following data items were also recorded for each of the eligible studies: author(s), year of publication, publication status (1 = published, 0 = unpublished), country in which the study was conducted, dominant religion of the country, gender composition (% of females), age composition (mean or median age), measure of religious coping, dimension of religious coping, measure of symptom, type of symptom, and language of the article. Any missing information, clarifications, or verifications were requested from the authors of individual primary studies through email.

The religion-related data were extracted from the GALLUP and NationMaster databases (69, 70). The numbers of confirmed COVID-19 cases and deaths reported during the study period were extracted from the Our World in Data database (71). The national data regarding the strictness of containment measures imposed during the study period were obtained from the database of the Oxford COVID-19 Government Response Tracker (63).

### 2.6. Statistical analysis

Many studies have examined and reported more than one effect size, and it is noteworthy that the problem of dependence could bias the standard errors and statistical inferences of a meta-analysis. This potential methodological problem was addressed using a three-level meta-analysis (72), taking into account the hierarchically nested structure of the present data.

A χ^2^-based homogeneity test was performed to assess heterogeneity across the eligible studies. The presence of between-study heterogeneity was indicated by a significant Cochran’s Q statistic (i.e., *p* < 0.05). The level of heterogeneity was indicated by the inconsistency index *I*^2^. In line with a widely adopted standard (73), heterogeneity was interpreted as (a) *no to low* for an *I*^2^ value that was less than 25%, (b) *moderate* for an *I*^2^ value that fell within the 25–75% range, and (c) *high* for an *I*^2^ value that was greater than 75% (73). A random-effects model should be used if the results indicate a significant moderate to high level of heterogeneity, and a fixed-effects model should be used otherwise.

Mixed-effects meta-regression analysis was used to test the moderation effects that might explain the between-study heterogeneity. The meta-regression was undertaken in a multivariate manner, with relevant predictors and control variables entered simultaneously (72). To unpack the significant moderation effects, simple slope analysis was conducted to estimate the effect sizes of the subgroups that were high (+1.5 SD) and low (−1.5 SD) in a particular dimension.

Outlier analysis was performed on the individual effect size estimates to detect possible outliers (74). If outliers were identified, the outlier removal method was used (75). The results yielded by this method were compared with those derived from the full dataset. If there were discrepancies, both sets of results were reported. If both sets of data yielded a highly similar pattern of findings, only the full findings were reported.

For the major analyses, both the three-level meta-analysis and meta-regression analysis were conducted using RStudio for Windows (version 2022.02.0+443; RStudio Team, Boston, MA) with the R package metafor (76). Krippendorff alpha was derived from the SPSS macro KALPHA (66) conducted in IBM^®^ SPSS^®^ (version 26.0; IBM Corp, Armonk, NY).

### 2.7. Study risk of bias and certainty assessments

The meta-analysis pooled the effect size estimates from an array of individual studies, and inevitably, the methodological quality varied vastly across the studies. The risk of study bias was evaluated using study quality assessment (77). The following indicators were coded and evaluated based on previous meta-analytic reviews (e.g., (78, 79)): statistical power (1 = Cronbach alpha ≥ 0.70, 0 = Cronbach alpha < 0.70), sampling method (1 = probabilistic sampling, 0 = non-probabilistic sampling), study design (1 = longitudinal, 0 = cross-sectional), measurement reliability (proportion of measures with adequate internal consistencies), and measurement validity (proportion of validated measures).

It is also important to detect the potential confounding effects of various biases and to verify the robustness of the study conclusions. To detect the possible risk of single-study bias, one-study-removed sensitivity analysis was conducted for all six effect size estimates. This sensitivity analysis examined whether the overall meta-analytic findings would be altered by the omission of any single eligible study. The robustness of the findings would be demonstrated if an overall effect size estimate remained stable across the various stages of study removal. However, significant changes in any of the effect size estimates indicated the presence of single-study bias.

Publication bias was estimated using various common statistical techniques. Specifically, potential publication bias was first visualized by a funnel plot, and then Egger’s linear regression test was conducted to identify funnel plot asymmetry (80). A significant test result revealed that the funnel plot was asymmetrical, suggesting the presence of publication bias. Finally, trim-and-fill techniques were used to adjust for missing studies, and the effect size estimates yielded after this adjustment were compared with those yielded by the original dataset (81). Publication bias was revealed if substantial differences were detected between these two effect size estimates. The sensitivity analysis and all the methods for publication bias assessment were performed using Comprehensive Meta Analysis version 2.2.020 (Biostat, Englewood, NJ).

Finally, *p*-curve analysis was used to assess the robustness of the present meta-analytic findings by estimating the true effect size (82). This analysis yielded curves that showed the distribution of *p*-values reported in the eligible studies, and the evidential value of the present pool of eligible studies was demonstrated by significant right-skewed *p*-curves.

## 3. Results

### 3.1. Study selection

**FIGURE 1:**
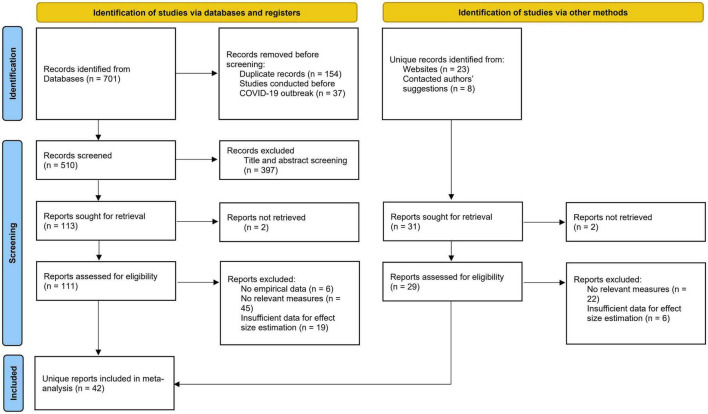
Flow diagram of search and selection strategies for reports in the present meta-analysis (64).

The literature search and coding stages involved in this meta-analytic review are summarized in a PRISMA flow chart (see Figure 1). After we applied the predefined eligibility criteria, the final dataset consisted of 42 primary studies. Table 1 summarizes the study characteristics of these studies included in the meta-analysis.

**TABLE 1:** Summary table of the sample and study characteristics of all 42 eligible articles.

References	*n*	Sample	Female %	Mean age	Country	Coping measure	Western religion
Agha (104)	100	Community-dwelling adults	17	33.13	Saudi Arabia	COPE	N
Bakır et al. (105)	327	Pregnant women	100	35.00	Turkey	RCOPE	N
Barbato (106)							
Sample 1	339	Community-dwelling adults	8	26.56	United Arab Emirates	RCOPE	N
Sample 2	204	Community-dwelling adults	24	41.59	United Arab Emirates	RCOPE	Y
Beato et al. (107)	428	Community-dwelling adults	75	40.80	Portugal	COPE	Y
Besirli et al. (108)	200	Healthcare workers	59	29.50	Turkey	COPE	N
Blackwell (109)	207	Undergraduates	80	19.32	United States	Others	Y
Chodkiewicz et al. (110)	618	Internet users	81	26.04	Poland	COPE	Y
Chow et al. (111)	200	Healthcare workers	61	36.00	Malaysia	RCOPE	N
Davis et al.(112)	184	Internet users	46	64.46	United States	RCOPE	Y
DeRossett et al. (45)	970	Internet users	43	38.43	United States	RCOPE	Y
Dobrakowski et al. (33)	365	Catholics	75	35.64	Poland	RCOPE	Y
Fallahi et al.(113)	183	Young adults	57	22.00	United States	COPE	Y
Fekih-Romdhane and Cheour (34)	603	SNS users	74	29.20	Tunisia	RCOPE	N
Fteropoulli et al. (114)	1,071	Healthcare workers	73	36.86	Cyprus	COPE	Y
Gökmen et al.(115)	823	Community-dwelling adults	38	26.00	Turkey	RCOPE	N
Gurvich et al. (116)	1,495	Community-dwelling adults	82	41.00	Australia	COPE	N
Hayes et al. (117)	319	Adults with gastrointestinal problems	25	40.37	Multiple countries	COPE	n/a
Kadiroğlu et al. (53)	514	High school students	53	16.97	Turkey	RCOPE	N
Lee (118)	775	Internet users	42	32.72	United States	Others	Y
Lee et al. (119)	398	Internet users	48	35.91	United States	Others	Y
Mahamid and Bdier (120)	400	Community-dwelling adults	57	32.00	Palestine	Others	N
Mahmood et al. (121)	408	SNS users	60	n/a	Pakistan	Others	N
Margetić et al. (122)	2,857	Community-dwelling adults	81	39.00	Croatia	COPE	Y
Mirhosseini et al. (123)	200	Patients with COVID-19	0	n/a	Iran	RCOPE	N
Mishra et al. (124)	588	University students	72	20.90	India	COPE	N
Ouanes et al. (125)	67	Elderly	46	65.50	Qatar	Others	N
Pačić-Turk et al. (126)	125	Healthcare workers	84	42.29	Croatia	RCOPE	Y
Prazeres et al. (127)	222	Healthcare workers	74	37.00	Portugal	Others	Y
Quiroga-Garza et al. (128)	604	Community-dwelling adults	78	41.89	Mexico	COPE	Y
Rababa et al. (129)	248	Elderly	42	63.95	Jordan	RCOPE	N
Salazar et al. (130)	677	Employees	50	48.75	Spain	COPE	Y
Shousha et al. (131)	199	Patients with COVID-19	54	43.79	Egypt	COPE	N
Singh et al. (132)	348	Healthcare workers	44	31.80	India	COPE	N
Skapinakis et al. (133)	3,379	SNS users	73	42.00	Greece	COPE	Y
Smida et al. (134)	127	Medical students	38	n/a	Qatar	COPE	N
Uvais et al. (135)	94	Patients	15	36.00	United Arab Emirates	COPE	N
Van Steenkiste et al. (136)	42	Healthcare workers	81	31.50	Belgium	COPE	Y
Vitorino et al. (137)	1,156	People under quarantine	70	37.60	Brazil	RCOPE	Y
Vitorino et al. (138)	1,156	Internet users	70	37.60	Brazil	RCOPE	Y
Yee et al. (139)	528	Community-dwelling adults	62	35.75	Malaysia	COPE	N
Yıldırım et al. (30)	255	Community-dwelling adults	88	32.96	Saudi Arabia	RCOPE	N
Zarrouq et al. (35)	1,435	Community-dwelling adults	43	32.20	Morocco	RCOPE	N

COPE, Religious Coping Subscale of the Coping Orientation to Problems Experienced Scale; N, no; n/a, not applicable or not available; RCOPE, Religious Coping Orientation to Problems Experienced Scale; SNS, social network site; Y, yes.

### 3.2. Study characteristics

Of the 42 primary studies, the majority (96%) were journal articles, 2% were research reports, and 2% were unpublished theses or dissertations. The pool of eligible studies comprised 43 independent samples with a total of 25,438 participants (mean = 592; SD = 674.57). In the samples, 58% were females, and the mean age was 36.50 years (SD = 10.67). The samples were drawn from 24 countries spanning seven world regions: 33% from Europe (e.g., Croatia and Spain), 26% from the Middle East (e.g., Palestine and the United Arab Emirates), 15% from North America (e.g., Mexico and the United States), 12% from Asia (e.g., India and Malaysia), 7% from South America (Brazil), 5% from Africa (Egypt and Morocco), and 2% from Oceania (Australia).

Western religion (Protestant Christianity, Roman Catholicism, Orthodoxy) was dominant in 47% of the countries, and non-Western religion (Islam and Hinduism) was dominant in 51% of the countries. The remaining 2% were studies that included more than two countries, and thus, the national religion-related data could not be coded. The average national religiosity score of the entire pool of eligible studies was 78.55 (SD = 16.17), and ranged from 32 (Australia) to 97 (Egypt).

### 3.3. Main-effect analysis of religious coping–symptom associations

Table 2 summarizes the results of the main-effect analysis of all the associations between the three dimensions of religious coping (i.e., GRC, PRC, and NRC) and psychological symptoms. As shown in the left panel of the table, there were significant positive associations between NRC and psychological symptoms (*p*s < 0.0004). In contrast, all the other religious coping–symptom associations were non-significant.

**TABLE 2:** Main-effect analyses and homogeneity tests for the associations between three dimensions of religious coping and psychological symptoms (*r*).

	Full data			Outlier removal data		
	**GRC**	**PRC**	**NRC**	**GRC**	**PRC**	**NRC**
**Main-effect analyses**						
Averaged *r*	0.0425	–0.0240	0.2886	0.0412	0.0304	0.2830
Lower 95% CI	–0.0547	–0.1331	0.1083	–0.0066	–0.0404	0.1128
Upper 95% CI	0.1398	0.0851	0.4689	0.0890	0.1013	0.4532
*k*	53	32	26	51	31	25
**Heterogeneity tests**						
*Q*	354.3542	388.5356	1242.9376	164.9939	301.0303	796.9080
*df*	52	31	25	50	30	24
*p*-value for *Q*	<0.0001	<0.0001	<0.0001	<0.0001	<0.0001	<0.0001
${\text{τ}}_{\left(2\right)}^{2}$	0.0000	0.0301	0.0049	0.0000	0.0194	0.0045
${\text{τ}}_{\left(3\right)}^{2}$	0.0400	0.0132	0.0712	0.0074	0.0000	0.0630

GRC, general religious coping; NRC, negative religious coping; PRC, positive religious coping.

The outlier analysis revealed four outliers. Two were identified in the studies that examined GRC, one in the studies that examined PRC, and one in the studies that examined NRC. Using the outlier removal method, the pattern of results was highly similar to that yielded from the full dataset, and the conclusions drawn from both sets of findings were the same (see the right panel of Table 2). Hence, the meta-analysis and moderation analysis were performed using the full dataset.

All the Cochran’s Q statistics indicated the presence of heterogeneity (*p*s < 0.0001), and all the *I*^2^ statistics indicated moderate levels of heterogeneity. These results demonstrated that the random-effects model should be used and that moderation analysis should be conducted to identify the sources of heterogeneity.

### 3.4. Moderation analysis

The results of the moderation analysis are summarized in Table 3. As shown in this table, there were some significant moderation effects for PRC and NRC, but all the moderation effects for GRC were non-significant. Two sample characteristics—age and sex composition—moderated the associations between PRC and psychological symptoms. Specifically, *post-hoc* simple slope analysis indicated that the hypothesized negative PRC–symptom association was marginally significant in samples with a greater proportion of older participants (*r* = –0.2015, SE = 0.1172, *p* = 0.0854), but this association was positive in samples with a larger proportion of younger participants (*r* = 0.4078, SE = 0.0743, *p* < 0.0001). Moreover, there was a significant positive PRC–symptom association in samples including more female participants (*r* = 0.2618, SE = 0.0800, *p* = 0.0011), whereas this association was non-significant in samples including fewer female participants (*r* = –0.0556, SE = 0.0888, *p* = 0.5315).

**TABLE 3:** Moderator analyses for the associations between three dimensions of religious coping and psychological symptoms.

	GRC (*k* = 48)		PRC (*k* = 30)		NRC (*k* = 26)	
**Characteristics**	***B***	**SE**	***B***	**SE**	***B***	**SE**
**Sample**						
Age composition	0.0108	0.0380	-0.2031**	0.0391	-0.0884	0.0377
Sex composition	0.0280	0.0478	0.1058**	0.0208	-0.0253	0.0151
**Religion-related**						
Dominant religion^a^	0.0340	0.0560	0.1157	0.0482	0.1110**	0.0388
National religiosity	-0.0532	0.0655	0.0812	0.0811	0.1735**	0.0407
**Socioeconomic**						
GDP per capita	-0.0089	0.0562	0.2018	0.1028	0.3209**	0.0288
Unemployment	-0.0327	0.0186	0.0248	0.0557	0.0176	0.0212
**COVID-19-related**						
Confirmed cases (per 100k)	0.0036	0.0333	-0.0323	0.2416	-0.0765	0.0796
Death cases (per 100k)	0.0095	0.0279	0.0532	0.0529	0.0730	0.0469
Containment strictness	0.0295	0.0168	-0.0753	0.0436	-0.1144	0.0616

GRC, general religious coping; NRC, negative religious coping; PRC, positive religious coping. ^a^1 = Western, 0 = non-Western. **p < 0.01.

Two national religion-related characteristics—dominant religion (Western vs. non-Western) and religiosity—were found to moderate the associations between NRC and psychological symptoms. The positive NRC–symptom associations were significant in countries with a non-Western dominant religion (*r* = 0.1668, SE = 0.0490, *p* = 0.0007) as well as in countries with a Western dominant religion (*r* = 0.4249, SE = 0.1857, *p* = 0.0221), but the magnitude of these significant positive associations differed. In addition, the hypothesized positive NRC–symptom association was significant in countries with higher religiosity (*r* = 0.6639, SE = 0.0679, *p* < 0.0001) but only marginally significant in those with lower religiosity (*r* = 0.1434, SE = 0.0749, *p* = 0.0556).

### 3.5. Risk of bias in studies

The results indicated that the majority of the eligible studies (91%) had adequate statistical power. Approximately two-thirds of the studies included measures that displayed adequate internal consistency (67%) and included validated measures (65%). However, only 7% of the studies used a random sampling method, and only 2% adopted a longitudinal or prospective design.

The results of the moderation analysis revealed that study design had significant moderating effects on the association between GRC and psychological symptoms (*B* = –0.1625, SE = 0.0289, *p* < 0.0001). Although the GRC–symptom association was non-significant in the majority of studies adopting a cross-sectional design (*r* = 0.0569, SE = 0.0537, *p* = 0.2892), it is noteworthy that the negative GRC–symptom association was marginally significant in the small subset of studies adopting a longitudinal or prospective design (*r* = –0.1265, SE = 0.0654, *p* = 0.0530).

Significant moderation effects of scale reliability were also found on the association between PRC and psychological symptoms (*B* = 0.1330, SE = 0.0502, *p* = 0.0081). The hypothesized positive PRC–symptom association was obtained in studies adopting less reliable measures (*r* = –0.2540, SE = 0.0992, *p* = 0.0104), but this association was non-significant in studies adopting more reliable measures (*r* = 0.1449, SE = 0.0866, *p* = 0.0942).

The one-study-removed sensitivity analysis revealed considerable stability for each of the six effect size estimates, indicating low risk of single-study bias. Regarding publication bias, the results revealed that the Egger’s tests failed to indicate publication bias (all *p*s > 0.05). Similarly, the trim-and-fill techniques did not alter the pooled effect size estimates. Finally, the *p*-curves of all three religious coping–symptom associations were significant and right-skewed, demonstrating considerable evidence value and the absence of the *p*-hacking problem. The statistical power of the three religious coping–symptom associations were also high, ranging from 0.96 to 0.99. Taken together, these analyses provided empirical evidence for the robustness of the meta-analytic findings.

## 4. Discussion

The present meta-analysis investigated the use of religious coping and its association with psychological symptoms experienced amid the initial wave of the COVID-19 pandemic, a period during which a series of unprecedented physical distancing measures were in place and public stress levels were heightened. The magnitude of the religious coping–symptom association was found to vary according to the dimension of religious coping. Overall, the hypothesized negative association between NRC and psychological symptoms was identified, but the associations with psychological symptoms were non-significant for both GRC and PRC.

Although the overall PRC–symptom association was found to be non-significant, the strength of this association was moderated by the age and sex composition of the samples. A significant positive PRC–symptom association was obtained in eligible studies including a higher proportion of younger participants and those including more female participants. It is noteworthy that younger individuals and women have been identified as two psychologically vulnerable groups amid the initial wave of the COVID-19 pandemic, with both women (vs. men) and the younger (vs. older) generation generally reporting higher levels of anxiety and depression (35, 51, 83–86). Both of these psychologically vulnerable groups may have a greater need to cope with their heightened distress, but the sole use of religious coping may not adequately mitigate their heightened levels of psychological symptoms. These findings did not reveal the hypothesized mental health benefits of PRC in mitigating psychological symptoms, but more frequent use of this type of religious coping was associated with higher levels of psychological symptoms during the first wave of the COVID-19 pandemic, especially for psychologically vulnerable people.

Previous research has unveiled two major cognitive mechanisms—cognitive reappraisal and perceived coping efficacy—that accounted for the mental health benefits of PRC (87, 88). These findings imply that the use of PRC as the sole strategy to mitigate stress-related distress may not be sufficient. In light of the previous findings, the use of PRC should be accompanied by the deployment of other adaptive cognitive and behavioral strategies, namely positive reframing, emotion regulation, and seeking social support (87). The hypothesized mental health benefits of PRC was not found in the first wave of the COVID-19 pandemic, a particular context when the etiology, treatment, and preventive measures were still unknown (89–92). In this period of high stress and uncertainty, many residents of COVID-19-affected regions reported a strong sense of helplessness and lack of control (93, 94). In addition, social isolation tends to decrease opportunities for these residents to receive support from social network members living apart (95). Such unusual circumstances and heightened stress levels may weaken the effectiveness of PRC in handling pandemic stress, which is largely beyond people’s control (93).

The present meta-analysis revealed robust findings regarding the hypothesized positive association between NRC and psychological symptoms. In the literature, three psychological mechanisms have been identified to account for this positive association: life stress, cognitive processes, and delay in seeking professional treatment (21). Public anxiety and stress reached unusually high levels during the initial wave of the COVID-19 pandemic. The use of problem-focused coping, such as doing regular exercise and practicing hand hygiene, has been found to be effective in reducing pandemic stress (96). Excessive attention to religious activities may enhance people’s tendency to neglect the deployment of these effective strategies; in particular, the use of NRC may lead vulnerable people to pay excessive attention to real or imagined “sin” (21). More importantly, excessive dependence on religious rituals and activities may delay professional treatment for mental health problems, which may lead to an aggravation of psychological symptoms (21). Hence, a “balanced” use of PRC together with other complementary adaptive coping strategies (e.g., cognitive reframing and emotion regulation) should be more effective in bolstering mental wellness during the COVID-19 pandemic (97).

Apart from demographic differences, national differences in the strength of the NRC–symptom association were also found in both the dominant religion (Western vs. non-Western) and religiosity, especially the latter. The positive NRC–symptom association was weaker in countries characterized by a Western religion and lower in religiosity. This intricate finding may reflect the recent observations in the decline in the level of religiosity in Western countries such as the United States (98). However, a stronger positive NRC–symptom association was found in countries with greater religiosity. This is probably because excessive attention to religious activities may increase one’s tendency to ignore work and family, which may in turn elicit additional sources of life stress such as job stress and family conflict (99). Also, excessive dependence on religious rituals and activities may delay the seeking of professional assistance for treating psychological problems, thus leading to more serious psychiatric disorders over time (21).

The present findings may have practical implications for mental health professionals. As religious coping is a preferred strategy for many people, facilitators of mental health treatment programs should make good use of PRC and also work to reduce the use of NRC when treating their clients who have experienced mental health issues during the COVID-19 pandemic (28, 97). As reviewed above, the use of religious coping may not be adequate for attaining this treatment goal, but clients need to acquire a variety of effective coping skills to broaden their coping repertoire (100, 101). For instance, facilitators may mitigate clients’ sense of helplessness by showing them how to distinguish between aspects of the pandemic that are under their control (e.g., perceived responsibility for one’s own health and adherence to health advice on COVID-19 prevention) and those that are beyond their control (e.g., implementation of mandatory disease mitigation measures and undesirable behaviors by others such as stockpiling). PRC should be used together with a number of complementary strategies to mitigate psychological symptoms and enhance mental wellness amid the COVID-19 pandemic (102).

In addition to practical implications, our meta-analytic findings also have implications for researchers. The null findings for the GRC–symptom association suggest that conceptualizing and assessing religious coping as a unidimensional construct should be avoided. Rather, the findings provide further evidence that PRC and NRC are conceptually distinct and thus should be assessed by separate scales, each with a unique set of coping behaviors. In addition, it is important to note that the mental health benefits of GRC were found in a small subset of studies that contained more than a single time point. In this small but significant body of longitudinal or prospective studies, GRC was found to reduce psychological symptoms over time during the COVID-19 pandemic. The overall null findings for the GRC–symptom association may have been yielded by the cross-sectional studies that currently dominate the literature on coping with COVID-19. More longitudinal or prospective studies should be conducted to capture the trajectory of the use of religious coping and an array of other coping strategies to deal with stress as the pandemic unfolds.

Finally, our study quality assessment revealed some methodological limitations in the studies included in the present meta-analysis. Very few (7%) of the studies relied on probabilistic sampling for participant recruitment, and even fewer (2%) investigated the use of religious coping over more than one time point; thus, possible changes in strategy deployment and mental health levels over time remain largely unknown. As public anxiety tends to fluctuate across various waves of a disease outbreak (103), more follow-up studies using probabilistic sampling methods should be conducted to address these methodological issues, thus advancing our understanding of when and how the use of religious coping can exert desirable and undesirable effects on the general public’s mental health.

## Data availability statement

The original contributions presented in this study are included in this article, further inquiries can be directed to the corresponding author.

## Author contributions

CC contributed to the study design, investigation, supervision and coordination of data coding and all other research tasks, statistical analysis, data interpretation, and wrote the first draft of the manuscript. WY contributed to the investigation, literature search, data interpretation, and wrote portions of the manuscript. Both authors contributed to the article and approved the submitted version.

## Footnotes

1
https://osf.io/shb32/
